# Surface Enhanced Raman Spectroscopy for DNA Biosensors—How Far Are We?

**DOI:** 10.3390/molecules24244423

**Published:** 2019-12-04

**Authors:** Edyta Pyrak, Jan Krajczewski, Artur Kowalik, Andrzej Kudelski, Aleksandra Jaworska

**Affiliations:** 1 Faculty of Chemistry, University of Warsaw, 1 Pasteur St., 02-093 Warsaw, Poland; epyrak@chem.uw.edu.pl (E.P.); jkrajczewski@chem.uw.edu.pl (J.K.); 2 Nencki Institute of Experimental Biology of Polish Academy of Sciences, 3 Pasteur St., 02-093 Warsaw, Poland; 3 Holy Cross Cancer Center, 3 Stefana Artwińskiego St., 25-734 Kielce, Poland

**Keywords:** SERS, DNA, surface enhanced Raman spectroscopy, nanosensor, biosensor, DNA mutations, thiolated DNA, nanoparticles, Raman reporters, label-free

## Abstract

A sensitive and accurate identification of specific DNA fragments (usually containing a mutation) can influence clinical decisions. Standard methods routinely used for this type of detection are PCR (Polymerase Chain Reaction, and its modifications), and, less commonly, NGS (Next Generation Sequencing). However, these methods are quite complicated, requiring time-consuming, multi-stage sample preparation, and specially trained staff. Usually, it takes weeks for patients to obtain their results. Therefore, different DNA sensors are being intensively developed by many groups. One technique often used to obtain an analytical signal from DNA sensors is Raman spectroscopy. Its modification, surface-enhanced Raman spectroscopy (SERS), is especially useful for practical analytical applications due to its extra low limit of detection. SERS takes advantage of the strong increase in the efficiency of Raman signal generation caused by a local electric field enhancement near plasmonic (typically gold and silver) nanostructures. In this condensed review, we describe the most important types of SERS-based nanosensors for genetic studies and comment on their potential for becoming diagnostic tools.

## 1. Introduction

There is strong interest in the development of new sensors for the sensitive identification of specific DNA fragments, for example, circulating free tumor DNA. Identifying circulating tumor DNA can be used to detect mutations in genes of predictive and prognostic importance for molecularly targeted therapies, and can therefore influence clinical decisions [1]. Currently, the most popular techniques for studying genetic predispositions for cancer are PCR (polymerase chain reaction) and NGS (next generation sequencing); however, the waiting time for the results of specific tests can be as long as 3 months, with a relatively high cost and a risk of false positive (contamination) or false negative (low sensitivity) results. Therefore, new methods are being developed to improve or even replace the routinely used techniques. Surface-enhanced Raman scattering (SERS) [2,3,4,5] is one promising ultrasensitive method that can be used to sensitively and accurately identify such specific DNA fragments. There are many different methods that can be used to detect specific DNA (usually containing a mutation) using SERS spectroscopy. In many SERS DNA sensors, a Raman reporter (a moiety having an exceptionally large cross section for Raman scattering, also called a label or tag [6]) is moved close to the plasmonic nanostructure, or removed from its proximity when a DNA fragment with a given sentence is present in the analysed sample [7,8,9]. The “movement” of the Raman reporter close to or away from the plasmonic nanostructure is realised by standard hybridisation (leading to a double-stranded DNA) or by the formation or decomposition of a hairpin chain (stem–loop configuration). Changing the distance between the Raman reporter and the plasmonic nanostructure (close or away) induces a significant change in the intensity of the measured SERS signal: the signal is very strong when the Raman reporter is close to the plasmonic nanostructure and weak when this distance is large. Another approach applied in SERS DNA sensors utilises the formation of agglomerates of plasmonic nanoparticles or plasmonic and magnetic nanoparticles induced by the presence of specific DNA fragments (in this case, the agglomeration is induced by hybridisation with the single-stranded DNA immobilised on the surface of the nanoparticles). It is also possible to carry out label-free SERS detection of a specific DNA by determining the structure of the linking moiety, by means of which the capture DNA is immobilized on the SERS substrate [10].

In this work, the most important types of SERS DNA sensors used to identify specific DNA fragments are briefly described. The basic theory of SERS enhancement, which is required to explain the mechanisms of the functionality of various SERS DNA sensors, is also presented.

### 1.1. Mechanism of Surface-Enhanced Raman Spectroscopy

The illumination of nanostructures made from metals with a negative real and small positive imaginary dielectric constant (for example, silver or gold nanostructures) leads to an excitation of the collective oscillations of surface conduction electrons known as surface plasmons. Surface plasmons may be treated as vibrating electric dipoles, which create a strong electric field in their proximity. For a spherical metal nanoparticle, the magnitude of the induced electric dipole (p) is proportional to [11,12,13,14,15]:

$$p\sim \frac{\epsilon_{M}\left(\lambda \right)-\epsilon_{out}\left(\lambda \right)}{\epsilon_{M}\left(\lambda \right)+2\epsilon_{out}\left(\lambda \right)}$$

where λ is the wavelength of the radiation-illuminating metal nanoparticle, and ϵ_M_(λ) and ϵ_out_(λ) are the dielectric functions of the nanoparticle and the surrounding medium, respectively. As can be deduced from the above equation, when the value of ϵ_M_(λ) becomes close to the value of −2ϵ_out_(λ), a very strong electric dipole is induced because the denominator of the fraction proportional to the magnitude of the induced electric dipole becomes close to zero. Inducing a large electric dipole leads to the local creation of a very strong electric field. The dielectric function of metal is a complex number, which means that it is not possible to satisfy the condition ϵ_M_(λ) = −2ϵ_out_(λ) exactly, and hence to obtain a situation where *p*→∞. The induction of a very strong electric field is observed when the imaginary part of ϵ_M_(λ) is small; in such a case, a so-called surface plasmon resonance is induced (for example, for silver nanostructures the surface plasmon resonance is induced when visible radiation is used) [16].

An increase in the intensity of the electric field induces an increase in the efficiency of various optical processes, including Raman scattering. Spectroscopy utilising the increased efficiency of Raman scattering generated by plasmonic nanoresonators is called surface-enhanced Raman scattering (SERS). The SERS enhancement factor (which measures the increase in the efficiency of the generation of the Raman signal) is roughly proportional to the fourth power of the electric field enhancement [17]. This fourth-power dependence makes it possible to obtain very large SERS enhancement factors, and in some cases it is even possible to record an SERS signal from a single molecule [13]. The very large achievable SERS enhancement factors make SERS spectroscopy one of the most sensitive analytical tools known.

The field enhancement generated by plasmonic nanostructures strongly depends on the distance from the surface of the plasmonic nanoresonator—the actual field dependencies are slightly different for various geometries, although it is often assumed that the SERS enhancement decreases as a function of r^−10^, with increasing distance from the plasmonic nanoparticle [15,18].

When the frequency of the excitation radiation is tuned to the absorption band of the analyte, the intensity of its Raman signal increases significantly. This effect is called resonance Raman scattering. Therefore, in addition to the above-described electromagnetic enhancement of the efficiency of the generation of the SERS spectra, the other effect, what is known as a charge transfer enhancement, which resembles the ordinary resonance Raman process, also contributes to the SERS enhancement. In the theory describing this effect, the assumption is made there is a hybridization of the orbitals of an adsorbed molecule with the orbitals from the metal. This allows resonance Raman scattering to occur, which leads in turn to an additional increase in the efficiency of the Raman signal generation (usually one to two orders of magnitude [19,20]). Such resonance enhancement is only observed for species directly interacting with the metal surface, which means that, although this enhancement is significantly smaller than the electromagnetic enhancement, in many cases it makes SERS spectroscopy very surface-sensitive (bands due to the vibrations of the molecules interacting directly with the metal surface are often the strongest ones).

### 1.2. Currently Developed Methods for Detecting DNA Mutations

Nowadays, a great research effort is being put into the development of techniques that make it possible to detect DNA mutations that are strongly related to the early diagnosis of genetic disorders [21], with the aim of improving the quality of data obtained [6]. Among those techniques, PCR (polymerase chain reaction) and its modifications (such as qPCR, quantitative polymerase chain reaction, and ddPCR, droplet digital PCR), BEAMing [22], different types of blotting (e.g., Northern blot or Western blot) or next-generation sequencing [23] are mostly used to detect DNA mutations as part of cancer treatment [1].

It is currently difficult to imagine detecting mutations in DNA and RNA without using the polymerase chain reaction (PCR) method. PCR is currently the basis for all commonly used genotyping methods. A comparison of currently used molecular biology and SERS methods for detecting mutations in DNA is presented in Table 1.

#### 1.2.1. Allele-Specific PCR

The first of the methods used was allele-specific PCR, also called an amplification refractory mutation system (ARMS-PCR), whose mutation detection principle relies on the specificity of the 3‘ end of one of the mutation detection primers. Two primers are used, one fully complementary to the mutated DNA sequence and another primer that is fully complementary to the non-mutated DNA sequence. Each pair is completed by a universal reverse starter. Two separate PCR reactions are performed to detect mutations. In the presence of a mutation, the use of a primer complementary to the mutated DNA and a universal reverse primer in the reaction will result in a band in the gel electrophoresis, while in the absence of a mutation, the band will not appear. Reactions with a pair of fully complementary primers are used as reaction controls. There are many variations of the allele-specific PCR technique. It was a technique that was once widely used, before technologies based on fluorescent dyes appeared. Agarose electrophoresis is required to visualize the bands [24,25,26].

#### 1.2.2. qPCR Quantitative Polymerase Chain Reaction (qPCR)

This is very simple, fast and cost-effective technique. This method utilizes two probes containing two different fluorophores (hydrolysis probe, which incorporates the use of a fluorophore and quencher), and one of the probes is complimentary to the wild sentence and the second to the mutated chain. During the PCR reaction, the probe is hydrolyzed during primer extension and the amplification of the specific sequence it is bound to. The cleavage of the probe separates the fluorophore from the quencher and results in an amplification-dependent increase in fluorescence. Thus, the fluorescence signal from a probe-based qPCR reaction is proportional to the amount of the probe target sequence present in the sample. Different fluorophores enable the detection of different alleles in the sample, i.e., wild type and, eventually, mutated ones, if present in the studied sample [27].

#### 1.2.3. PCR-HRM (High Resolution Melt)

This method is based on real-time monitoring of PCR amplification, applying intercalating fluorescent dye. The dyes have a unique property; they bind specifically to double-stranded DNA and they fluoresce brightly in that state. In the absence of double-stranded DNA, they can’t bind and the fluoresce is at a low level. After PCR temperature is increased gradually, which causes DNA double-stranded melting, the dye dissociates from denaturated, single-stranded DNA. Detecting small differences in PCR amplicon melting (dissociation) curves enables differentiation the melting plots and detection mutation in the amplified DNA molecules. Then, wild-type and heterozygous samples can be easily differentiated in the melting plots [28,29].

#### 1.2.4. Sanger Sequencing

The first step is to perform a PCR reaction with ddNTP (dideoxynucleotides), which are incorporated into the synthesized DNA strand by a polymerase, but their incorporation leads to termination, i.e., prevents further chain extension. This is because ddNTP does not have a 3’-hydroxyl group. Sequencing takes place using ddNTPs labeled with various fluorochromes that are titrated to non-fluorescently labeled dNTPs. During PCR, both ddNTP and dNTP are incorporated into the synthesized DNA chain randomly and termination only occurs when ddNTP is incorporated. As a result, DNA fragments of different lengths are created, each of them terminated with ddNTP labeled with appropriate fluorescence and complementary to the DNA of the template. During capillary electrophoresis, DNA fragments are separated and arranged from the longest to the shortest, which allows the DNA sequence that constituted the template to be read [26].

#### 1.2.5. ddPCR

The ddPCR method is based on emulsion-droplet technology, thanks to which a single real-time PCR mixture is fractionated in 20,000 droplets (emulsion in oil) in real-time. In the next step, prepared emulsion is termocycled and then each droplet is scanned, enabling sensitive and precise detection of the presence of even a single DNA molecule in each droplet. The system reads the target (positive) and all other (negative) molecules, based on which the absolute number of target molecules in the sample, are generated, without having reference standard curves or a reference gene (as opposed to qPCR). The ddPCR is very sensitive technology and it is widely adopted in liquid biopsy for druggable mutation detection or real time monitoring of treatment in patient’s plasma samples [30,31,32].

#### 1.2.6. NGS

Next-generation sequencing (NGS) is a term used to describe a number of different modern DNA and RNA sequencing technologies. Most of these technologies are based on a massive sequencing of short pieces (100–200 bp) of DNA and RNA molecules. NGS allow for sequencing of DNA and RNA much more quickly (>1,000,000,000 x) and cheap comparing to the previously used Sanger sequencing. Currently available sequencing technologies can be divided into two types due to the length of the sequenced molecules:

(a) short DNA sequences, usually between 100 and 400 bp in length (IonTorrent and Illumina technology).

(b) long sequences of DNA/RNA, currently between several thousand and over 100 thousand bp in length (Nanopores).

The basis of most NGS technologies currently available on the market (Illumina, San Diego, California, U.S. or IonTorrent, Gilford, New Hampshire, U.S.) is mass sequencing of short DNA sequences (from 100 to 400 base pairs), i.e., several million reads from several million short DNA fragments are obtained. Sequencing of very long, naturally occurring DNA segments is developing intensively. These are native DNA or RNA molecules that only require preparation in the form of attachment of priming sequences and proteins that will enable the start of sequencing, as is the case with nanopore technology. Currently available technologies allow sequencing of the entire human genome within a few days. Nowadays, the most common application of NGS in tumor diagnostics is panel sequencing of 10–300 genes in one test from the sample. NGS transforms scientific research and genetic diagnostics of cancer in particular [33,34].

PCR was first described in 1983, and uses the ability of DNA polymerases and flanking oligonucleotide primers to efficiently and precisely replicate a targeted stretch of DNA during each cycle, so that the number of copies of a sequence of interest increases exponentially, thus providing enough material for downstream analysis [24]. With modern thermocyclers, this can be achieved quickly and with minimal hands-on time. Nowadays, qPCR dominates the diagnostics market at 70%, but it requires expensive fluorescent dyes intercalating in DNA or probes (short DNA sections) with intercalating dyes attached. This significantly increases the cost of testing. The qPCR method has a sensitivity of 1%–5% of the mutation frequency in the sample. Other methods used in this type of research are Sanger sequencing (with a sensitivity of about 10%–20% of the mutated allele, but which detects all mutations in the tested sequence), other PCR variants (e.g., ASA-PCR, HRM-PCR), and the intensively developing next generation sequencing (NGS), which identifies all mutations and has a very high sensitivity of 0.1%–5%. Unfortunately, NGS is currently a multi-stage, complicated method, and is therefore time-consuming and very expensive).

Each of these methods has a different detection limit, cost, analysis time and specificity. PCR-based tests detect a limited number of known mutations. However, the sensitivity of these methods is about 1%–5% of the mutated allele [25]. The accuracy of these methods depends on the amount and quality of DNA extracted from tissue [26]. Therefore, some mutations may not be detected, due to sample contamination, disseminating necrosis, or too little available DNA. Hence, the role of molecular research in oncology all over the world is gradually increasing, due to the introduction of new molecularly targeted drugs for treatment.

Surface-enhanced Raman spectroscopy offers a good alternative for studies on DNA mutations, due to its very low limit of detection, and usually clear and easy to interpret results [27,28]. Here, we summarize different approaches for SERS-based nanosensors for DNA studies, and critically discuss their potential in medical applications as an alternative to PCR and the other methods described above. Since the DNA Raman sensors have not yet been applied in the clinical applications, the cost of the individual analysis is unknown.

## 2. Characteristics of SERS-Based Sensors—General Approach

The model detection of point DNA mutations by means of SERS spectroscopy can usually be performed by two experimental procedures: with the use of Raman reporters and label-free detection. The main advantage of direct detection is its experimental simplicity; however, with complex samples, the signal may be indistinguishable and too low for the reliable detection of, for example, one base mutation. Therefore, Raman reporters are often included in SERS-based DNA experiments, enabling the indirect detection of DNA. The greatest advantage of this methodology is that it features a much higher cross section for the Raman scattering of Raman reporter molecules compared to DNA. Therefore, in this review we will focus mainly on examples of SERS sensors including the use of labels, although we will also examine the most interesting examples of label-free detection for comparison.

### 2.1. Label-Free Sensors

**Figure 1:**
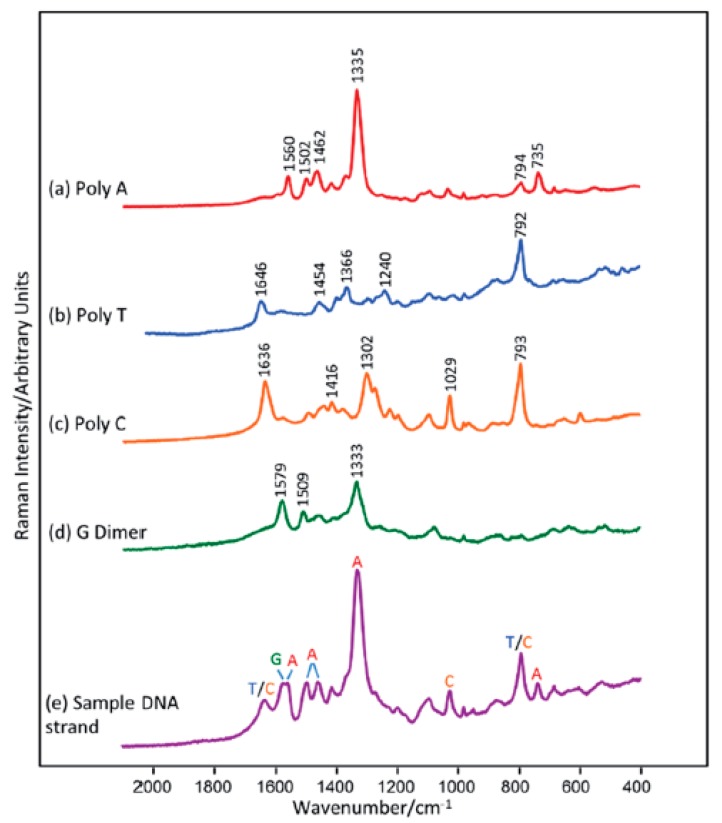
SERS spectra of the four main DNA nucleotides, (a) polyadenosine, Poly A, (b) polythymidine, Poly T (c) polycytosine, Poly C and (d) guanosine dinucleotide, G dimer, (e) the SERS spectrum of a ss-DNA sequence: CTT–TTT–CCT–GCA–TCC–TGT–CTG–GAA–G. Reprinted from reference [41] with permission from the Royal Society of Chemistry. Copyright 2017 RSC.

**Figure 2:**
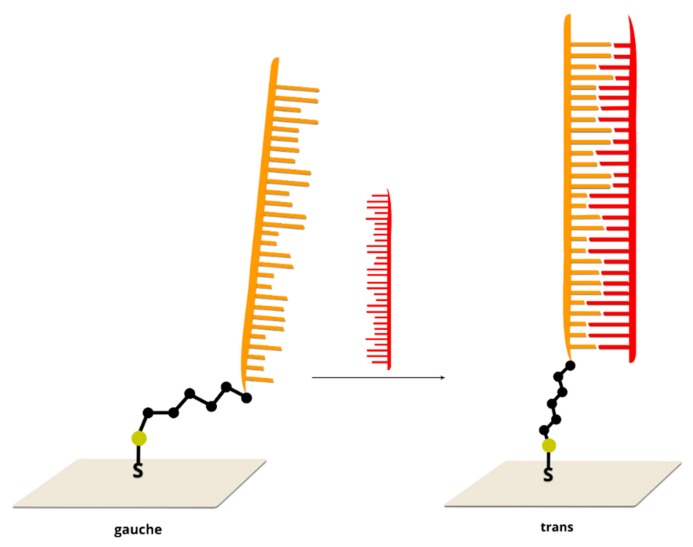
Scheme of label-free sensor based on spectral changes induced by changes in the orientation of the DNA strand on the surface after hybridization [10]; orange is capture DNA immobilized on the metal surface and red is the target DNA.

**Figure 3:**
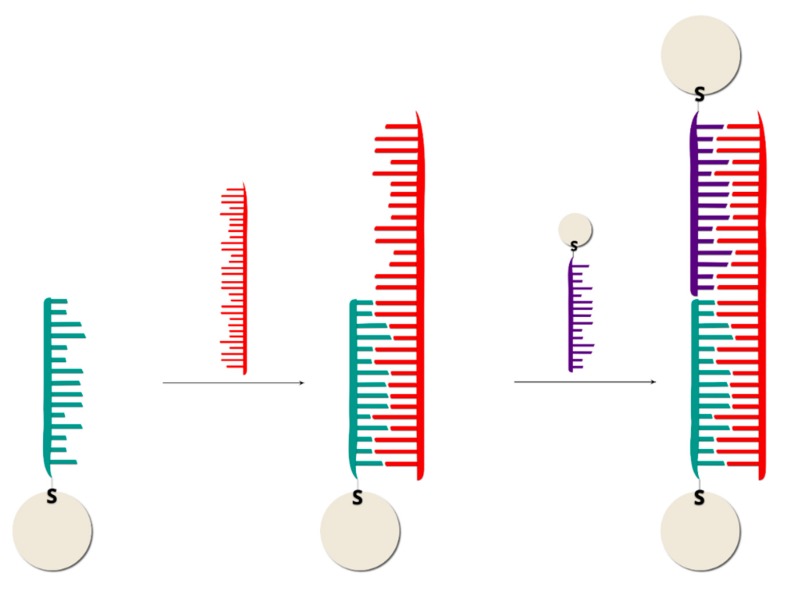
Scheme of label-free sensor: green is capture DNA immobilized on nanoparticles, red is the target DNA (for example, mutated DNA from a clinical sample), and violet is complementary DNA immobilized on nanoparticles.

Label-free (without using Raman reporters) DNA detection is much easier in terms of the preparation of the sample process, although the SERS signal of the DNA (or moieties modifying the DNA) is much weaker than when a Raman reporter is used. However, there are interesting examples of DNA detection in a direct way [29,30,31,32,33,34,35,36,37,38]. Usually, the SERS signal depends on the quantity of DNA strands in the sample and the content of specific bases in the DNA chain, sometimes even with a one base difference sensitivity—see Figure 1. [39,40]. However, usually the changes in the SERS spectra because of the hybridization process (due to changes in conformation, charge, interaction with the surface distance, etc.) are the background for label-free SERS detection of DNA [9,41]. For example, hybridization can cause conformational changes (a *gauche*→*trans* of the linking layer, see Figure 2) in alkanethiol-modified DNA immobilized on a metal surface, which can be seen by comparing the relative intensity of the specific bands in the SERS spectra [10]. The experimental procedure in this case requires only the attachment of a specific alkanethiol-modified ssDNA to the metal surface, the addition of complimentary ssDNA, and the SERS measurement. However, the Raman cross section of the linking layer by which the DNA is attached to the metal surface may be not high enough to provide a reliable tool for clinical applications of label-free SERS, since even a small change in the DNA sequence can drastically alter its orientation on the surface [42]. In another label-free experiment, although one that requires a more complicated experimental procedure (similar to the application of labels), three ssDNAs are hybridized. Briefly, thiolated single-stranded DNA with a specific sequence is attached to plasmonic nanoparticles or plasmonic and magnetic nanoparticles [43,44,45,46,47,48,49,50]. Due to hybridization, in the presence of the target DNA, nanoparticles form agglomerates (Figure 3), which can be easily detected from the increase in the intensity of the measured SERS signal in the case of plasmonic agglomerates (the SERS enhancement factor is especially large for species located in the narrow slit between plasmonic structures), or from the formation of the plasmonic-magnetic agglomerates, which can concentrate by the magnetic field [51]. The formation of the agglomerates indicates the presence of the target DNA.

In most cases, the SERS spectrum of the DNA is dominated by a strong signal of adenine at 736 cm^−1^. The substitution of adenine with 2-aminopurine in the probe DNA sequences can be used to detect complementary sequence hybridization and discrimination against non-specific DNA binding [31]. The SERS spectra of 2-aminopurine substituted DNA are significantly different from those of adenine consistent DNA. The most important, 2-aminopurine has no band at 736 cm^−1^. Therefore, DNA hybridization could be identified by the presence of a 736 cm^−1^ adenine band. The quantitative determination of DNA hybridization is based on the ratio of the intensity of the 736 cm^−1^ adenine band and that of the 807 cm^−1^ 2-aminopurine band. The intensity of the 2-aminupurine band is constant and depends only on the DNA packing density. In the case of no hybridization, the ratio of these bands is close to zero and achieves maximum value for complete hybridization of the target and probe DNA.

It is well known that adenine exhibits a higher Raman cross-section than other nucleotides [52]. This phenomenon can be used to detect mutations in the BRCA-1 gene sequence [32]. The SERS spectra of DNA are highly concentration-dependent. At high concentrations, the spectra are dominated by the adenine ring breathing mode at 736 cm^−1^. This band significantly decreases at lower concentrations. Additionally, the skeletal ring vibrations in the spectral region between 1200 and 1600 cm^−1^ are more intensive. This could suggest that the orientation of the adenine base with respect to the metal surface changes from perpendicular to flat as the concentrations decrease [53]. Interestingly, the SERS spectra of thiolated DNA containing only adenine moieties (polyA) at low concentrations are identical to those of unthiolated polyA. As unthiolated polyA can only bind non-specifically via the nucleobase, this suggests that, at low concentrations, thiolated polyA also binds through the sulphur atom to the silver surfaces. Spectral changes related to increasing concentrations are associated with conformation change. At high concentrations, DNA strands are attached only by a thiol liver and then stand up from the surface. In order to detect a mutation in the BRCA-1 gene, ssDNA with a stem and loop structure was attached to the metallic surfaces. The DNA fragment in the loop is complementary to part of the BRCA-1 gene sequences. Hybridization leads to the opening of the loop, and, hence, the double-stranded DNA stands up from the surface. Such an orientation leads to an enhancement of the adenine ring breathing mode at 736 cm^−1^. For non-complementary sequences, hybridization does not occur and only a small band at 736 cm^−1^ is observed.

Typically, the direct SERS analysis of unmodified DNA is limited to single-strand DNA. Direct interaction between double-stranded DNA and nanostructured metal surfaces (negatively charged at physiological pH) is highly impeded by the negative charge of the phosphate backbone. This results in poor spectral reproducibility and limited sensitivity. The solution to this problem is to apply appropriately charged silver nanoparticles functionalized by spermine [54]. Spermine molecules improve the stability of the nanoparticles and, after the addition of DNA, promote the aggregation of the nanoparticles into stable clusters. SERS spectra are collected from a colloidal suspension with a long-distance objective, which results in a record of statistically averaged results from a large volume. Such a system makes it possible to acquire SERS spectra of two complementary DNA strands and their corresponding double helix structures. In this experiment, the overall dsDNA concentrations were kept at 630 ng/mL, whereas the vibrational assignment of the recorded signal was based on the literature references. It was found that the carboxyl stretching mode at 1653 cm^−1^ is extremely sensitive to disruption of the Watson–Crick hydrogen bonds. Such a system could be used to detect single-base mismatches. Subtraction of the SERS spectra of native and mismatched DNA generated difference spectra containing vibrational signatures associated with additional (positive features) and a removed nucleobase (negative features). For example, in the case where adenine was replaced by guanine, negative bands appeared at 1487 and 1577 cm^−1^, ascribed to purine modes, while the other spectral region did not exhibit any differences.

A similar approach, based on silver nanoparticles functionalized by a spermine molecule, was also used by another group [55]. It was observed that the addition of either single strands or duplexes induced a nanoparticle aggregation into stable clusters, mediated by the electrostatic interaction between the negatively charged phosphate backbones and the positively charged metallic surface. The SERS spectra recorded exhibited strong bands at 599 cm^−1^ (cytosine ring bending mode), 684 cm^−1^ (guanine ring breathing modes), 733 cm^−1^ (associated with adenine) and 1089 cm^−1^, ascribed to the symmetric stretching of the phosphodioxymoiety, ν(PO_2_^−^). The band at 790 cm^−1^ contains two distinct components due to the cytosine (C) and the thymine (T) ring breathing vibrations. Therefore, it is possible to extract the relative thymine content from the composed C + T ring breathing band at ~792 cm^−1^ once the initial C content is determined via an analysis of the 599 cm^−1^ feature. Again, as an internal standard, the intensity of the ν(PO_2_^−^) band at ~1089 cm^−1^ was used. This permitted a quantitative analysis of the relative numbers of nucleobases in the DNA strands.

Large-scale gold nanoplate (GNP) films presented by Bi and co-workers are an interesting platform for label-free detection [56]. Authors have presented rapid and simple methods of their preparation and have tested this surface with SERS measurements of DNA bases. Obtained spectra were similar to the literature and have contained characteristic peaks of each base.

As mentioned above, the main challenge in the direct SERS analysis of unmodified DNA is connected to the negatively charged phosphate backbones, which impede DNA adsorption. Another problem is interference from the surfaces’ inference [57]. In the case where DNA containing 12 identical adenines was mixed directly with silver nanoparticles, the measured SERS spectrum was dominated by citrate ions. One possible solution is to coat the surface of the nanoparticles with an iodide monolayer. Such a monolayer not only cleans the surfaces, but also provides a strong chemical interaction between the metal surfaces and the biomolecules [33]. Also, the risk of denaturation is reduced. A further addition of MgSO_4_ neutralizes the surface charge and enhances the interaction of the DNA with the silver nanoparticles. In the case where only MgSO_4_ was added, the SERS signal was still dominated by citrate. After an iodide-only modification, citrate ions were removed from the nanoparticle surfaces; however, no DNA signal could be recorded. This was caused by an adverse negative charge of the nanoparticle surfaces. Mg^2+^ ions played a dual role, acting as a bridge between the nanoparticles and the DNA, and causing nanoparticle aggregation, which led to the formation of hot-spots. An analysis of the recorded SERS spectra of four 12-base homopolymeric DNA sequences made it possible to experimentally assign the observed bands to nucleotides. All the DNA strands exhibited a common band at 1087 cm^−1^ that could be assigned to the symmetric stretching vibration of the PO_2_^−^ moiety. For specific DNA sequences, the number of PO_2_^−^ groups was the same, and therefore it was possible to use the signal at 1087 cm^−1^ as an internal standard for a quantitative analysis of the number of each type of base in the DNA.

Mg^2+^ ions were also used with hydroxyloamine reduced silver colloids [33]. An analysis of the SERS spectra showed that colloid aggregation by using concentrated NaCl leads to an absence of DNA Raman signals, because the DNA cannot compete with the high salt concentration on the nanoparticle surface. MgSO_4_ does not bind strongly with the silver surface, and therefore allowed DNA sequences to bind through its constituent bases. This makes it possible to record the SERS spectra of unthiolated homopolymers or mononucleotides under suitable conditions. Each base yields characteristic bands that can be used to identify the nucleotides. Because the SERS spectra of the unthiolated DNA strands contain features associated with all of the constituent bases, it should be possible to detect even single changes in the DNA sequences. Such an analysis is based on a digital subtraction of the recorded spectra that should remove the contribution of unchanged nucleotides. Using this method, it is possible to detect A→G and C→A polymorphism in 25 and 23 mers DNA sequences.

As described before, the addition of an aggregating agent such as magnesium ions or spermine leads to an increase in the measured SERS signal. However, it was found that when negatively charged silver colloids are aggregated by Mg^2+^ ions, it is not possible in the SERS signal to differentiate between single-stranded and duplexed DNA [34]. Moreover, the SERS spectra of homopolymers and ssDNA are highly dependent on the composition of the nucleobase. In case of spermine-aggregated nanoparticles, such dependencies are almost cancelled out. Additionally, the removal of the external aggregating agent from the system greatly simplifies the sensing scheme and drastically reduces the number of variables, which should be carefully controlled.

It was found that dichloromethane can be used as an improving agent molecule to enlarge the distance between two spherical silver nanoparticles [35]. It can also be used as an internal standard based on its typical Raman band at 700 cm^−1^ assigned to the symmetric CH_2_Cl_2_ stretching vibration. This is possible because this band does not overlap with any characteristic peak of DNA nucleotides. It was found that the introduction of dichloromethane leads to an almost 20-fold increase in the measured Raman signal, in comparison to that without dichloromethane. Such a method makes it possible to detect the mutation of CGC into CAC, in which case the intensity of the typical band for G located at 655 cm^−1^ decreases, while the intensity of the other band related to A at 732 cm^−1^ increases. Other spectral alternations were also observed: the peaks for C and T decreased, due to the much lower SERS activity of these bases when base complementary pairing occurs. Such a mutation can be detected, regardless of whether the mutation is located at the front, the middle or the end of the ssDNA sequences.

**Figure 4:**
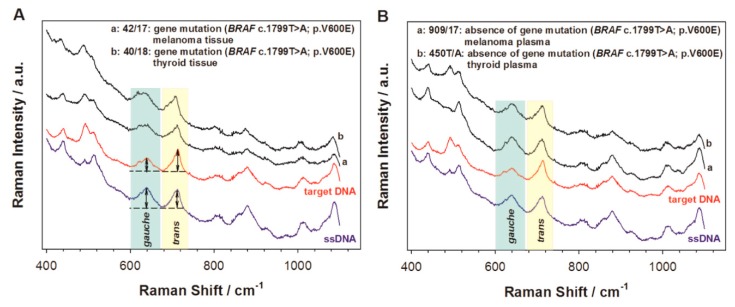
SERS spectra of ssDNA (blue lines) after interaction with DNA isolated from FFPE tumor tissue, with detected mutation (**A**), DNA isolated from plasma patients without detected mutation (**B**) and non–complementary synthetic DNA. Reprinted from reference [10] with permission from Elsevier. Copyright 2019 Elsevier.

Another method is based on the orientation of 5’ end thiolated ssDNA strands. It was found in the samples analyzed that the conformation of the hexanethiol linker, by which the DNA is attached to the surface, depends on the presence of ssDNA fragments complementary to the immobilized strands [10]. The SERS spectra exhibited two strong, characteristic peaks at 640 cm^−1^ and 714 cm^−1^. These bands could be associated with the ν(C–S) stretching vibration of the alkanethiols on the gold surface. The ν(C–S) band at the lower wavenumber (640 cm^−1^) is characteristic for the *gauche* conformation of the Au–S–C–C chain, while the band at the higher wavenumber (714 cm^−1^) is characteristic of a molecule having a *trans* conformation. In the case of the presence of only single DNA strand, the intensity ratio of the ν(C–S) band of the *trans* and *gauche* conformer was equal to 0.9. After hybridization with complementary ssDNA strands, the relative surface concentration of the hexanethiol moieties having a *trans* conformation of the Au–S–C–C chain significantly increased and reached a value above one. In the case where non-complementary DNA fragments were added, hybridization did not occur, and the band ratio did not change. Such a SERS sensor was successfully applied to detect circulating tumor DNA fragments. During the experiment, 17 different clinical samples were examined: 10 samples with BRAF mutation and seven samples without BRAF mutation. The samples with BRAF mutation contained ssDNA complementary to capture ssDNA. It was found that, in the clinical samples with complementary DNA fragments, the intensity ratio of the ν(C–S) bands of the *trans* and *gauche* conformer was equal to at least 1.04, while, for the samples that did not contain ssDNA complementary to the capture ssDNA, this ratio was equal to a maximum of 0.96. the recorded SERS spectra of clinical samples are shown in Figure 4. Therefore, the observed process of the rearrangement of the structure of the linkage moiety through which the capture ssDNA is attached to the metal surface may be used as an indicator of hybridization.

It is well known that ssDNA can adsorb on metal surfaces in randomly coiled conformations. It was found that, where DNA strands are modified by an NH_2_ external linker, the latter acts as a major anchoring point [58] and, as a result, the polybases are much more ordered, with a less random orientation than in the case of NH_2_-free polybases. It was found that, in the absence of an external NH_2_ linker, the polybase chains are adsorbed with a more or less coiled conformation on the Ag surface, while the DNA chains with an external NH_2_ linker may adopt a more linear and extended conformation. A possible explanation for this phenomenon is that the NH_2_ external linker acts as a strong mechanical anchoring point for the DNA strands. This leads to a reduction in the SERS intensity fluctuations. Such a solution makes it possible to record SERS spectra for polybase DNA fragments with characteristics bands for each of the nucleotides: 735 cm^−1^ for polyA, 792 cm^−1^ for polyC, 656 cm^−1^ for polyG, and 799 cm^−1^ for polyT.

A very interesting DNA sensor is based on reduced graphene oxide (RGO) functionalized by silver nanoparticles [59]. The reduced graphene oxide sensor generates a relatively weak SERS signal, because the chemical enhancement in SERS is significantly lower in comparison with the electromagnetic enhancement derived from the silver nanoparticles. Additionally, the SERS signal detected on the silver nanoparticles is weak, due to the weak interaction between the silver surface and the ssDNA chains. The graphene oxide and silver nanoparticle composites are an excellent platform for the SERS detection of DNA. This is caused by a noncovalent binding affinity between the DNA strands and the graphene sheets, where the ssDNA sequences lie directly on the graphene layer, with all the nucleotides flat, favouring π–π stacking between the bases and the aromatic carbons in the graphene. Therefore, the SERS signal in AgNPs@RGO DNA sensor is superior to the other two substrates, due to the synergistic effect of the electromagnetic enhancements and the strong interaction between the DNA and the graphene. A uniform distribution of silver nanoparticles on the graphene sheets guarantees high signal reproducibility. What is more, trapped DNA sequences can be washed away from the substrate and the sensor regenerated for further use. Typically, the SERS spectra recorded exhibit bands at 832, 1244 and 1513 cm^−1^ (assigned to polyC) and at 610, 359, 1407 and 1559 cm^−1^ (relayed to polyG). Moreover, the characteristics for graphene D and G bands do not overlap with the spectral signature of the DNA.

An interesting sensor for DNA detection with high selectivity and a low detection limit was proposed by Zhang and co-workers [60]. In such a sensor, in the first step, a DNA probe complementary to a segment of the target DNA was attached to magnetic microspheres through the carboxyl groups on the magnetic nanospheres and amino groups on the amine-functionalized DNA strands. The silver nanoparticles were functionalized by a p-mercaptobenzoic acid monolayer. After functionalisation, the silver surface was full of carboxyl groups, and, subsequently, a second DNA chain complementary to the target DNA was attached to those groups. The detection of the targeted DNA sequences was performed by mixing functionalized magnetic microspheres with the functionalized silver nanoparticles and the targeted DNA. Due to DNA hybridization, magnetic microspheres with silver nanoparticles were formed, and it was possible to isolate these from the solution by means of a magnet. The isolated magneto-plasmonic microspheres were dispersed in a Tris buffer, and SERS spectra were recorded. The analysis was based on the intensity of the peak located at 1077 cm^−1^. It was found that the sensor described exhibited a linear response over a wide concentration range of from 10 to 500 nM.

**Figure 5:**
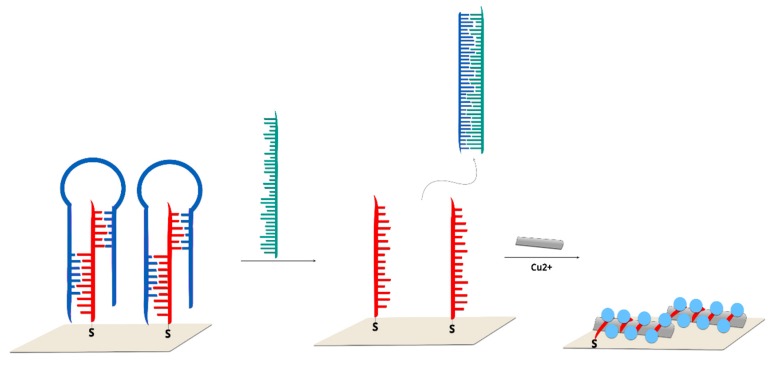
A scheme of SWNT-based SERS assay coupling with RNase HII-assisted amplification for highly sensitive detection of ctDNA in human blood [61]; blue is hairpin capture DNA immobilized on nanoparticles, red is the T-rich DNA, green is circulating tumor DNA, grey is single-walled carbon nanotube, and light blue are copper nanoparticles.

Label-free but no direct detection of DNA was presented by Zhou et al. [61]. In this approach, the observed 1605 cm^−1^ band in SERS spectrum was from G-band of single-walled carbon nanotubes (SWCNs). The triple-helix molecular switch (THMS) structure, which was used as recognition element, was formed from thymine-rich single-stranded DNA and capture DNA complementary to cancer tissue DNA immobilized on gold film. After RNase II enzyme-assisted amplification, T-rich ssDNA was adsorbed onto the surface of SWCNs. The presence of Cu^2+^ ions indicates the growth of CuNPs (Figure 5). The presented sensor allows femtomolar detection of DNA from the blood sample.

As mentioned above, unmodified DNA strands form a randomly coiled configuration due to different affinities of the various parts of nucleic acid to the gold or silver surfaces [62]. However, it was found that phosphate backbone groups can strongly interact with alumina nanoparticles [63]. Therefore, replacing gold nanoparticles with alumina ones prevents the non-specific adsorption of DNA chains on surface, and hence allows for a quantitative, label-free detection of DNA.

On the other hand, some critical reports suggest that the SERS method is not viable for the detection of DNA hybridization [64]. Their authors suggest that, because of the lack of spectral differences before and after hybridization, it is not possible to distinguish between hybridization and non-specific binding. Additionally, the limit of effective SERS detection is less than 5 nm from the metallic surface, indicating that the hybridization assay is sensitive only for the few first nucleotides.

In contrast to the above work, Lierop et al. demonstrated an elegant method of directly detecting PCR products using the difference in adsorption of ssDNA and dsDNA [65]. They observed that the signal from uni-molecular SERS primers was significantly lower than that from bi-molecular SERS primers.

### 2.2. Typical Sensors with Labels

Although DNA can be directly detected on plasmonic materials by SERS, the structural similarities of different sequences oligonucleotides result in spectra that are difficult to distinguish. A successful solution to this problem is the application of molecules called Raman “reporters” (RR) in SERS systems used for DNA detection.

As mentioned above, a Raman reporter is a molecule with an exceptionally large cross-section for Raman scattering. It can be present in an SERS sensor by attachment to ssDNA or adsorption on the metal surface. A strong signal from the Raman reporter in an SERS spectrum indicates the presence of the target DNA in the analysed sample. This is an indirect method of DNA detection, because we don’t obtain any information about the target DNA sequence or structure. There are a few different types of SERS sensors with labels: a “sandwich” structure, where one DNA strand is tagged with a Raman reporter; a structure where RR is located directly on the nanoparticles; and a type where the DNA with RR form a structure called a “hairpin” before or after hybridization with another DNA strand. Below, we describe each type of sensor, and give examples of sensors representing each category. Afterwards, we describe examples that, in our opinion, are worthy of mention, although they do not fit strictly into any of these categories.

#### 2.2.1. Sandwich Sensors with RR Connected to DNA

**Figure 6:**
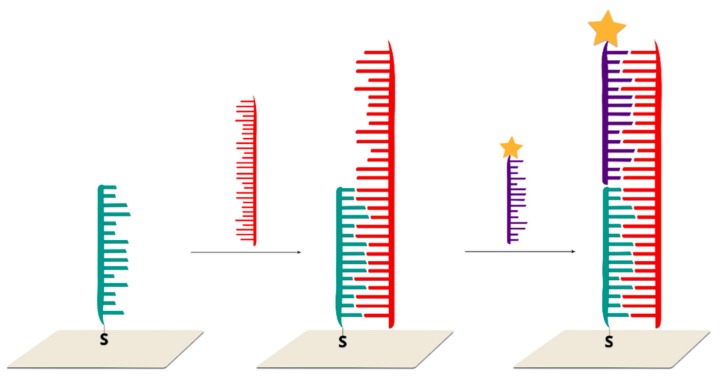
Scheme of sandwich type sensor: green is capture DNA immobilized on the metal surface, red is the target DNA (for example, mutated DNA from a clinical sample), and violet is complementary reporter DNA labelled with an RR.

The structure of a sandwich biosensor is quite simple. Thiolated, single-stranded DNA is adsorbed on a plasmonic surface (a roughened electrode, nanoparticles, etc.) and, in the next step, it hybridizes first with the target DNA (from the sample) and then with DNA labelled with an RR. A diagram of such a DNA sensor is shown in Figure 6.

**Figure 7:**
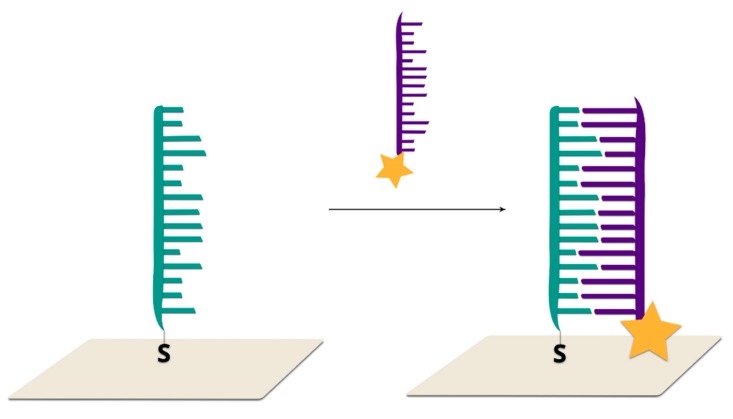
A prototype of sandwich type sensor consisting of two DNA strands by Vo-Dinh and co-workers [66]; green is capture DNA immobilized on the metal surface and violet is the target DNA labelled with an RR.

A prototype of the sensor based on this structure, but consisting of only two DNA strands, was described in 2003 by Vo-Dinh and co-workers [66] (Figure 7). In this approach, ssDNA with a thiol moiety on the 5′ end is immobilized on a silver surface with the formation of an Au–S bond. Next, 6-mercapto-1-hexanol is added to cover the empty spaces and avoid unspecific DNA binding. Capture DNA is hybridized with complementary strand labelled with rhodamine B on the 5′ end. This structure permitted the detection of BRCA1 breast cancer gene. A very similar approach, but with three different DNA strands, was applied for BCL2 gene detection, suggesting the reproducibility of this experimental approach to detect different mutations [67].

Another sensor build of two DNA strands had a similar structure, but a Raman reporter (Cy5) is added to the 3′ end [68]. After hybridization, the Cy5 is located in close proximity to the metal surface. A very similar approach for three different DNA strands was described by other groups [69,70,71], showing the relative versatility of this methodology.

A similar but more advanced structure was presented by Cao and co-workers [72]. Thiolated DNA labelled with Cy3 was immobilized on an Au nanoparticle surface and then mixed with the target DNA and capture DNA adsorbed on a microarray chip. To obtain higher enhancement, an Ag solution was added and Ag particles were grown around the Cy3-labelled nanoparticle probes. Not only Cy3 dye, but also Cy3.5, Cy5, TAMRA, Texas-Red and rhodamine 6G were tested in this work. System verification was performed on six oligonucleotide sequences: hepatitis A virus Vall7 polyprotein gene (HVA), hepatitis B virus surface antigen gene (HVB), human immunodeficiency virus (HIV), Ebola virus (EV), variola virus (smallpox, VV), and Bacillus anthracis (BA) protectiveantigen gene. This approach was later developed by immobilizing different, labelled oligonucleotides, mixed in a different molar ratio, on the nanoparticles [73], and authors assumed that by mixing different RRs, large numbers of characteristic spectroscopic signals can be obtained.

Another example of a three-strand “sandwich” structure was shown by He et al. [74]. First, thiolated single-strand capture DNA was immobilized on silver nanoparticles. The structure was then incubated with the target DNA and reporter DNA tagged with Rhodamine 6G. SERS spectra of the whole sensor were recorded, indicating the presence of the target DNA by the signal from the Rhodamine 6G. It is worth noting that the silver nanoparticles were first embedded on silicon nanowires (SiNWs). Silicon materials have many attractive properties: photocatalytic activity, high electrical conductivity, and antimicrobial effect. Moreover, the SiNWs@AuNPs structure analysed in this work gives a giant SERS enhancement factor of up to 10^10^. This high enhancement makes it possible to detect DNA at concentrations of about 1 fM and a one-base mismatch. Silicon was also used in a SERS substrate in the form of Si-poly(MeO_2_MA)-SH@Au in a “sandwich” sensor for the detection of the hepatitis B virus DNA [75], and as silica wafers decorated with silver nanoparticles [76].

**Figure 8:**
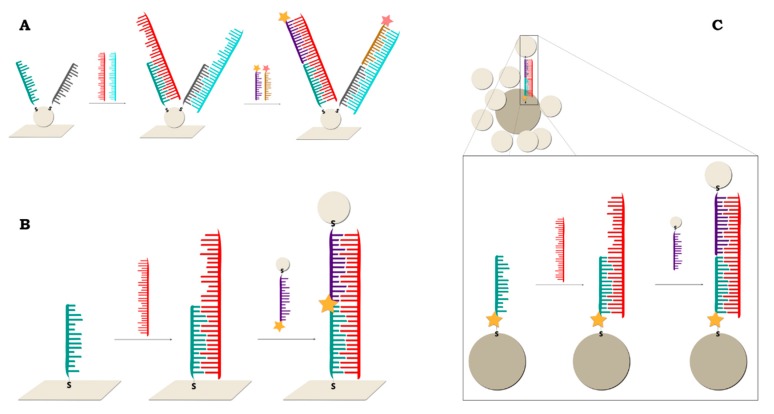
Different modifications of sandwich type of sensors based on: (**A**) the gold nanoparticle-decorated chemical vapor deposition (CVD)-growth graphene [77], (**B**) the Au particle-on-wire system [81]; (**C**) the core-satellite Au nanoparticles structure [78]; green and grey are capture DNA immobilized on the metal surface, red and light blue are the target DNA (for example, mutated DNA from a clinical sample), and violet and brown are complementary reporter DNA labelled with an RR.

A high enhancement of the Raman signal was also obtained in a biosensor built on gold nanoparticle-decorated chemical vapor deposition (CVD)-growth graphene [77] (Figure 8A). In this paper, two different sensor models were presented. The first structure was identical to the one described above, and the other was based on the same sensor structure but with more than one capture ssDNA strand immobilized on the metal surface. This made multiplex DNA detection possible—the simultaneous detection of many types of target DNA on the same substrate is possible.

A biosensor can also be composed of two sizes gold nanoparticles [78] (Figure 8C). A large nanoparticle acts as a core for smaller particles (satellites). Thiolated ssDNA is attached to the satellite particle, while the core strand is thiolated ssDNA with Cy5 dye located in the DNA strand next to the thiol moiety. Hybridization occurs due to the linker strand, which has parts complementary to both the core and satellite strands.

Silver nanorice antennae, coupled with a patterned gold triangle nanoarray chip, has been also used for a sandwich structure to detect hepatitis B virus DNA [79]. It turned out that a structure consisting of an Au triangle nanoarray chip, coupled with DNA and Ag nanorice labelled with an RR, made it possible to detect the target DNA even below 0.05 fM.

Au-coated paramagnetic nanoparticles can be also used as an alternative SERS surface for DNA detection [80]. This approach provides a satisfactory detection limit (at 20 nM), while the washing and collection of nanoparticles using a magnet can greatly facilitate the experimental procedure.

A similar approach, based on two metal materials as a platform for DNA immobilization, was presented by T. Kang et al. [81] (Figure 8B). An Au particle-on-wire system was constructed by the self-assembly of Au NPs onto Au NW through DNA hybridization. Both the target DNA immobilized on the gold nanowire and the reporter DNA with Raman dye immobilized on the gold nanoparticle were thiolated. To construct the final “sandwich” structure, the target DNA on the Au NW was incubated with the target DNA and then mixed with a solution of Au NPs connected to the labelled DNA. This system was applied for the diagnosis of clinical pathogens.

A typical sandwich assay, but with the use of encoded materials, was described by Qin et al. [82]. In this work, arrays of nanodisks prepared by on-wire lithography were used as a platform which was then functionalized with RR and oligonucleotides. This approach enabled DNA to be detected at an impressive level of 100 fM. These studies were continued, to improve the encoding capacity of the platform by the addition of silver [83].

A very interesting experimental approach was described by Hu et al. [84]. Capture and reporter DNA strands were designed to join many nanoparticles together forming a “tree-like” structure. The detection of HIV-1 DNA was at an impressive level of 10^−19^ M, although the specificity was perhaps not satisfactory, as the HIV-1 DNA signal was only 27% higher than the blank, while for the single base mismatch it was 10% higher.

Another example of atypical, promising nanostructures used as a DNA sensor are well-ordered, flower-like gold nanostructures; with them, however the sample concentration of around 1 μM is not yet good enough to implement this surface for lab-on-chip DNA analysis [85].

A somewhat complicated experimental approach involving the melting of dsDNA was described by Mahajan et al. [86,87]. In this approach, resonance SERS spectroscopy was used to monitor the denaturation of dsDNA attached to a gold surface. This made it possible to distinguish between a wild type, a single point mutation (1653C/T), and a triple deletion (DeltaF 508) in the CFTR gene at the 0.02 aM level.

It is worth mentioning that the majority of the capture DNAs used in SERS-based sensors are modified with a thiol linker. However, biotinated or amine-modified oligonucleotides can also be used [88], and they are a much cheaper alternative to thiol modification.

#### 2.2.2. Sandwich Sensors with RR Located on Nanoparticles

This model is similar to the classic “sandwich” described above, except that the Raman reporter is not attached to the reporter DNA strand but to the plasmonic nanoparticles. Reporter DNA is tagged with the same nanoparticles (Figure 8). After the complete construction of the sensor structure, the Raman spectrum of the whole system is recorded, and when a strong signal is obtained from the RR, this indicates the presence of the target DNA in the analysed sample—as in the previous type of sensor.

**Figure 9:**
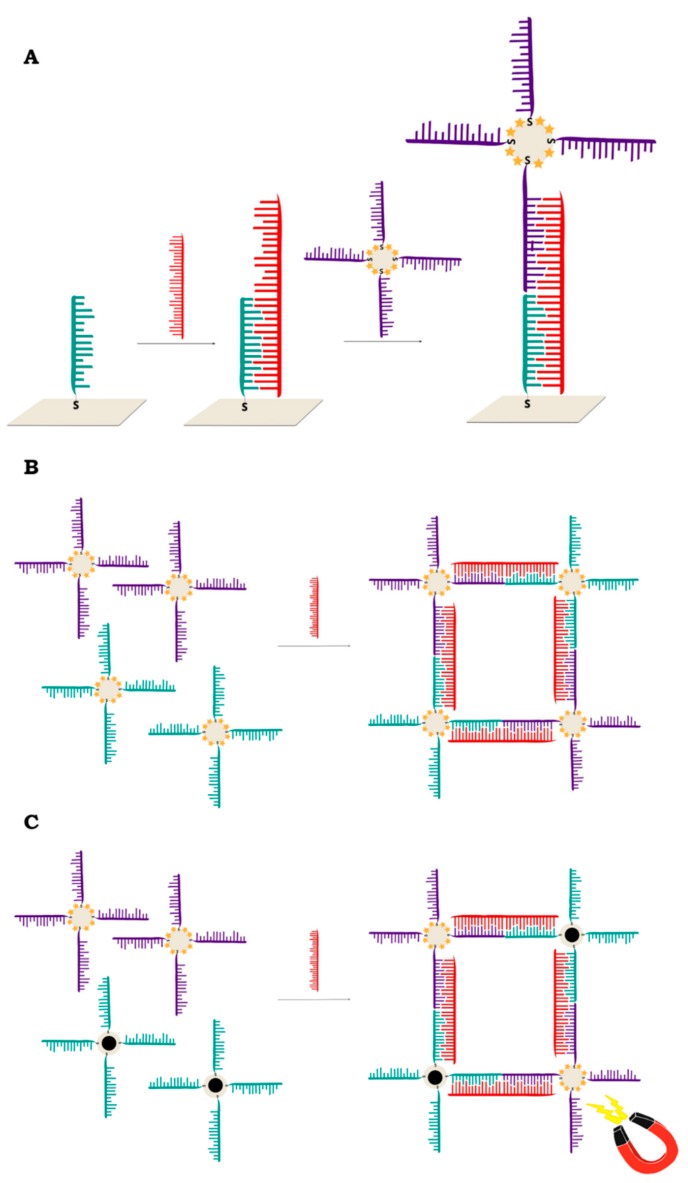
(**A**) General scheme of sandwich sensor with RR located on nanoparticles; (**B**) Scheme of DNA conjugates with dye-coded nanoparticles [89]; (**C**) Scheme of DNA conjugates with dye-coded and magnetic nanoparticles [44]; green and violet are capture DNA immobilized on magnetic nanoparticles or nanoparticles labelled with RR and red is the target DNA (for example, mutated DNA from a clinical sample).

Graham and co-workers have used dye-coded nanoparticles for the first time to detect DNA in conjugates where a signal was only achieved when hybridization has taken place [89] (Figure 9B). The nanoparticles were modified with 3,5-dimethoxy-4-(60-azobenzotriazolyl)phenol and functionalized with different, non-complementary thiolated oligonucleotides. The addition of target DNA complementary to both strands resulted in hybridization and the formation of conjugates. This structure was later modified by the addition of silver-coated magnetic nanoparticles [44] (Figure 9C).

Jaebum Choo’s team presented a few modifications of a sensor where malachite green isothiocyanate was adsorbed on gold nanoparticles with detection DNA and then hybridized with the target DNA and capture DNA (modified with biotin and streptavidin). Two of their works have applied to lateral flow strips, where this structure has been used as an alternative to the typical oligonucleotide-labelled AuNPs [90,91]. Their sensor was able to detect two DNA markers simultaneously.

An interesting structure is a multicolour Au–Ag nano-mushroom sensor for DNA detection [92,93]. Gold nanoparticles were covered with Raman reporters (4-aminothiophenol (ABT) or 4-nitrothiophenol) and capture DNA, which was, at the same time, the capture DNA for the sandwich structure and the gap DNA for the silver ‘head’ of the nano-mushroom. The structure of the sensor permitted the simultaneous detection of different DNAs in one sample due to various reporters, while the bimetallic structure (gold head and silver cap) ensured great enhancement. The authors have also described another strategy where DNA was labelled with an RR.

The first multiplex (four-plex and later eight-plex)) detection of DNA targets using nonfluorescent Raman tags was described by Sun et al. [94,95]. In this approach, thiolated ssDNA was immobilized on gold nanoparticles and then mixed with one of four R-tags: 4-mercaptopyridine, 2-thiazoline-2-thiol, 4,6-dimethyl-2-pyrimidinethiol or 2-thiouracil. At the same time, a gold-coated glass slide was covered with capture DNA and 6-mercapto-1-hexanol (to reduce nonspecific binding). Both of them hybridized the target DNA (breast cancer susceptibility gene 1) and were treated with a solution leading to the deposition of silver nanostructures. The authors demonstrated the first proof-of-concept study for applying SERS-based detection with non-fluorescent labels to investigate alternative gene splicing.

The multiplexed SERS detection of DNA was performed on core–shell nanospheres [96]. Poly(styrene-co-acrylic acid) nanospheres about 170 nm in size were covered with significantly smaller 20 nm silver nanospheres. Raman reporters were used: 4-aminothiophenol (4-ABT), 4-hydroxythiophenol (4-HBT), 4-chlorothiophenol (4-CBT) and 5,50-dithiobis(2-nitrobenzoic acid) (DTNB). Each of them had a sulphur functional group with a strong affinity for the silver surface. The RRs formed self-assembled monolayers on the Ag nanoparticle surfaces and were stabilized with silane coupling agents. The next modification of this system was the addition of TEOS in order to obtain a silica shell around the whole structure, which was then incubated with probe DNA. Capture DNA, ending with an amino group, was immobilized on the modified glass slide. The addition of the SERS probe constructed earlier and the target DNA resulted in a final SERS sensor being obtained.

A slightly different experimental approach was shown by Braun et al., where capture DNA was immobilized on a smooth metal film together with the RR, so that the SERS signal could not yet be recorded, and then, during the hybridization process, the target DNA, together with the reporter DNA immobilized on the silver nanoparticles, formed a structure with a much larger cross section for Raman scattering, as the RR was located between the silver nanoparticles and the flat silver film forming a silver sandwich structure with the dye inside [97].

A two-platform sensor based on gold nanoparticles was presented by Khalil and co-workers [98]. Graphene oxide-gold nanoparticles with immobilized thiolated capture DNA and complementary target DNA were deposited as the first element, whereas the second element was built from capture DNA and Cy3 adsorbed on the gold nanoparticles. A strong local enhancement was obtained after the hybridization of these two components. Even for the target DNA at a concentration of 10 fM, this structure was able to detect the difference in the analysed DNA with a precision of a single nucleotide.

Magnetic nanospheres have also been employed for DNA detection at the picomolar level [45]. Li et al. attached probe DNA (targeted to one part of the target ssDNA) to the nano-SERS-tags (poly(styrene-co-acrylic acid)/(silver nanoparticles)/silica composite nanospheres), and capture DNA (targeted to the other part of the target ssDNA) to the Fe_3_O_4_/poly(acrylic acid) core/shell nanospheres [45]. The nano-SERS-tags with probe DNA were first allowed to undergo hybridization with the target ssDNA in solution to achieve the best efficiency. Subsequently, the magnetic composite nanospheres with capture DNA were added as capturing substrates of the target ssDNA combined with the nano-SERS-tags. Upon attraction with an external magnet, the nanospheres (including the nano-SERS-tags) were deposited together due to the hybridization, and the deposit sediment was then analysed by SERS.

Target DNA, obtained directly from cancer cells, was detected in the sandwich system with non-fluorescent Raman labels [99]. This DNA was received on the way of RNA isolation from cancer cells and S1 nuclease digestion. To produce a sensor, thiolated capture DNA was immobilized on gold-coated glass slide covered with a silicone mask with an array of holes and hybridized with target DNA and probe DNA strand with AuP-RTag. This strategy has allowed the quantification of splice variants obtained directly from cells without amplification.

**Figure 10:**
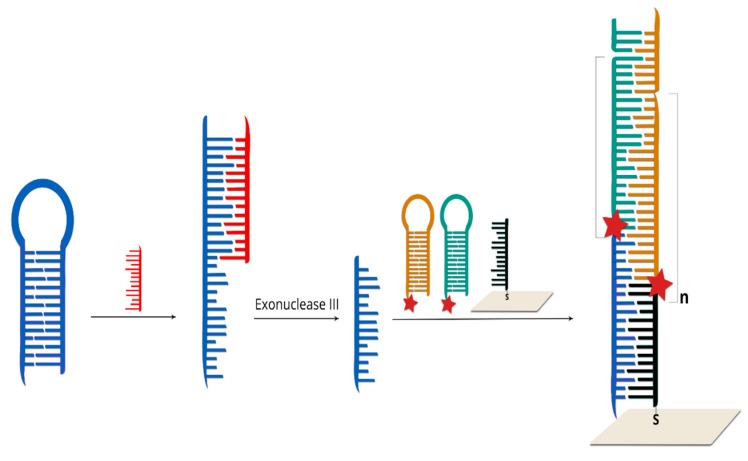
Schematic illustration of the Exo III-fueled target recycling-programmed cascade reactions integrated with SERS Technology for DNA Detection [102]; blue is hairpin probe DNA, red is the target DNA from clinical sample, black is capture DNA immobilized on the metal surface, green and brown are hairpin DNA labelled with RR.

Due to improvements in the limit of detection, sometimes SERS spectroscopy can be combined with amplification methods such as rolling circle amplification [100,101], Exo III-boosted cascade signal amplification [102] (Figure 10), Ligase Detection Reaction [103] or target-triggered enzyme-free amplification recycling [104].

#### 2.2.3. Hairpin On-Off Sensors

**Figure 11:**
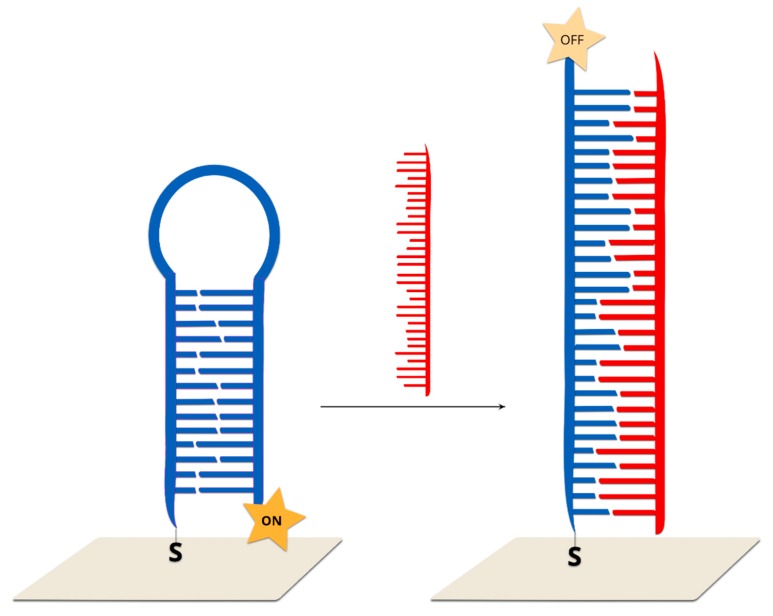
General scheme of hairpin on-off sensor: blue is hairpin capture DNA immobilized on nanoparticles labelled with RR, red is the target DNA (for example, mutated DNA from a clinical sample).

Single-stranded DNA, with one end tagged with a Raman reporter and the other end modified with thiol, is covalently bonded to a SERS-active structure. The immobilized DNA forms a hairpin chain, where the Raman reporter is located in close proximity to the plasmonic surface. This system generates a very strong SERS spectrum. After hybridization between the immobilized DNA and complimentary target strand, which disrupts the stem–loop configuration, the Raman reporter is separated from the surface of the plasmonic structure, which causes a decrease in the measured SERS signal of the Raman reporter (Figure 11).

Such a sensor was presented by X. Wei and co-workers. In this case, thiol-modified Cy5 tagged stem-loop DNA strands were self-assembled on gold nanoparticles immobilized on a silicon nanowire array. In a previous work, the enhancement factor for this SERS substrate was estimated at 10^6^–10^8^ [105]. Due to the small distance between the Cy5 molecules and the gold nanoparticles, strong Raman spectra could be observed. After DNA hybridization with the target DNA, the stem–loop configuration is disrupted and converted into rigid linear DNA. This leads to an increase in the distance between the Raman reporter molecule and the metallic surface, which, in turn, leads to a decrease in the measured signal. The described sensor has a good linear response in a wide concentration range from 1 nM to 10 pM. The analysis was based on the intensity of the prominent Raman peak at 1366 cm^−1^, which is assigned to the methine chain deformation of Cy5. Upon the addition of non-complementary DNA chains, the SERS intensity does not change. It was found that this method could be also used for single-base mismatch discrimination. In the case of fully complementary DNA, the Raman peak at 1366 cm^−1^ has a much lower intensive than in the case of a single-base mismatch.

A similar approach was tested in the Vo-Dinh group for the detection of single-base polymorphism in different genes [30,106,107]. Briefly, the SERS substrate was functionalized with capture thiolated DNA labelled with RR. The hybridization of this DNA with the complementary target DNA leads to a change in the distance between the dye molecule and the plasmonic surface to 35 nucleotides, and, hence, to a decrease in the intensity of the measured SERS signal.

This approach was also adopted by Qi et al. to successfully perform measurements in a microfluidic system with a target DNA concentration of 20 pM and only 10 minutes of analysis time [108], as well as by Kim et al., who used nanodendrites as SERS substrates and achieved a femtomolar detection limit [109].

A hairpin on–off system could be used to detect erbB-2 and ki-67 breast cancer biomarkers [110]. For this purpose, ERBB2 and KI-67 nanoprobes were modified by two different Raman labels, Cy3 and TAMRA, at the 5’ end, and by a thiol group at the 3’ end. During the experiments, appropriately high concentrations of MgCl_2_ were maintained because it was found that magnesium ions can stabilize the double-stranded stem regions. It was also found that the SERS intensity of both nanoprobes increased with Mg^2+^ ions concentrations and reached a maximum value at a concentration of 5 mM. After the addition of complementary targeted DNA, the stem–loop structure opened. Therefore, the distance between the Raman reporter molecule and the metallic surface increased by 10 nm (35 nucleotides). In such a case, the SERS intensity of the major Raman bands decreased significantly. In the absence of complementary DNA, or in the presence of non-complementary DNA, the SERS intensity of the major Raman bands remained high. Due to the presence of two different dye probes, the simultaneous detection of both biomarkers was possible. A similar method of Ki-67 gene detection was proposed by Wang and co-workers [111]. In their work, stem–loop DNA chains modified by TAMRA were also used, but a triangle-shaped silicon wafer with a high electromagnetic field coefficient was used as the SERS substrate.

A similar method could be also used for the screening of point mutation in cancer cells [112]. For this purpose, the KRAS gene was detected, which has been commonly found to contain mutations in cancer cells. This gene controls the process of cell proliferation through on and off switching in cell signalling. The mutation in the KRAS gene was found to be correlated with colorectal, pancreatic and lung cancer, and so identifying it could be used to differentiate cancer cells from healthy cells. DNA samples were extracted from two model cancer cell lines, MDA-MB-435 and SW480. Only the SW480 cells contained the mutated KRAS gene. The probed DNA chains were thiolated at the 3’ end and modified by FAM dyes at the 5’ end. As in the previous cases, in the absence of the target DNA, the probed DNA strand formed a stem–loop structure, and the FAM molecules were located on the metallic surface. In the presence of the target DNA, the hybridization of wild type KRAS with the probed DNA led to the opening of a stem–loop structure. During this transformation, the SERS signal decreased due to an increase in the distance between the Raman dye molecules and the nanoparticles. This method makes it possible to detect samples containing various amounts (10%, 50% and 90%) of mutations. It was found that the SERS intensity is negatively correlated with the proportion of the wild-type KRAS gene.

Based on a hairpin on–off system, it is also possible to detect HIV-1 DNA sequences [106]. It was found that proper DNA sequences consist of 42 base pairs, which form a six-base pair stem–loop structure. A Raman reported molecule Rhodamine 6G was applied. In the absence (blank) or presence of non-complementary target DNA sequences, the hairpin conformation remained stable, resulting in the proximity of Rhodamine 6G with the metallic surfaces, and a strong SERS spectrum was observed. In the presence of complementary HIV-1 target DNA chains, the stem–loop conformation opened, resulting in the physical separation of the dye molecule from the plasmonic surface and a significant decrease in the SERS signal. It was found that this SERS quenching effect increased with an increase in HIV-1 DNA concentrations.

An alternative method was proposed by Guo et al. [113]. It is well known that DNA can be attached to a metallic surface via an Au–S bond or by the intrinsic affinity of polyadenine for a gold surface. It was found that, by adjusting the length of the polyA chains, it was possible to change the conformation of DNA from ‘‘lying down’’ to ‘‘standing up’’. It is obvious that such a transformation leads to a change in the distance between another DNA end and the gold surface. Therefore, after labelling one DNA end with a Raman reporter molecule, it was possible to conduct in situ monitoring of the conformation transformation by SERS measurements. It was found that similar behaviour could be observed after the formation of double-strand DNA. Such a sensor exhibits a wide range of linear responses, from 0.1 to 500 nM, with an estimated detection limit of 45.7 nM.

**Figure 12:**
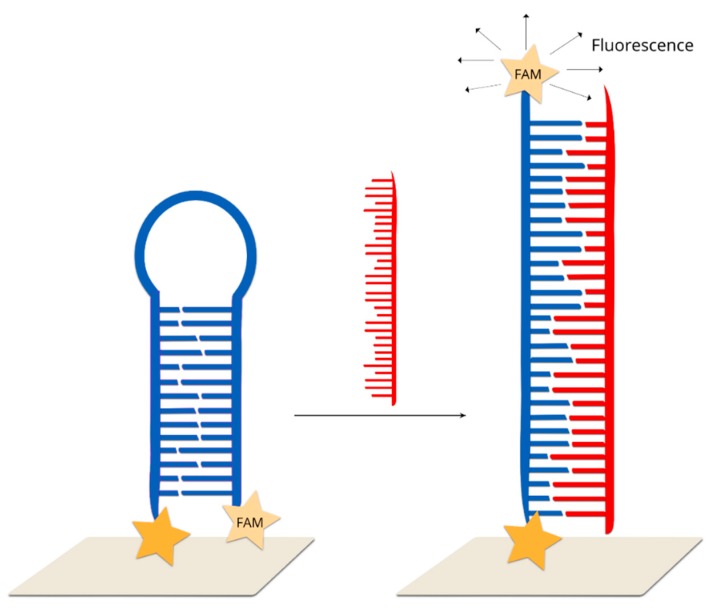
Example of hairpin on–off sensor with RR and fluorescent dye (FAM). Blue is hairpin capture DNA immobilized on nanoparticles labelled with RR and FAM, red is the target DNA (for example, mutated DNA from a clinical sample) [113].

A dual labelled probe containing an FAM dye at the 5′ terminus and a benzotriazole dye at the 3′ terminus was immobilized on silver nanoparticles in the form of stem–loop structure [114]. In this approach, resonance SERS technique was combined with fluorescence. Without a complementary target sequence, no fluorescence was detected and the SERRS spectrum was dominated by FAM peaks, while after hybridization, the hairpin structure was open, and the fluorophore was removed from the surface. After the hybridization, SERRS spectrum was dominated by benzotriazole bands and fluorescence could be emitted (Figure 12).

Another stem–loop DNA labeled with two fluorophores at the 5′ (TET) and 3′ (TAMRA) termini was used in PDMS microfluid channel for DNA detection [115]. In this approach, SERS was used to provide DNA hybridization.

#### 2.2.4. Hairpin Off-On Sensors

**Figure 13:**
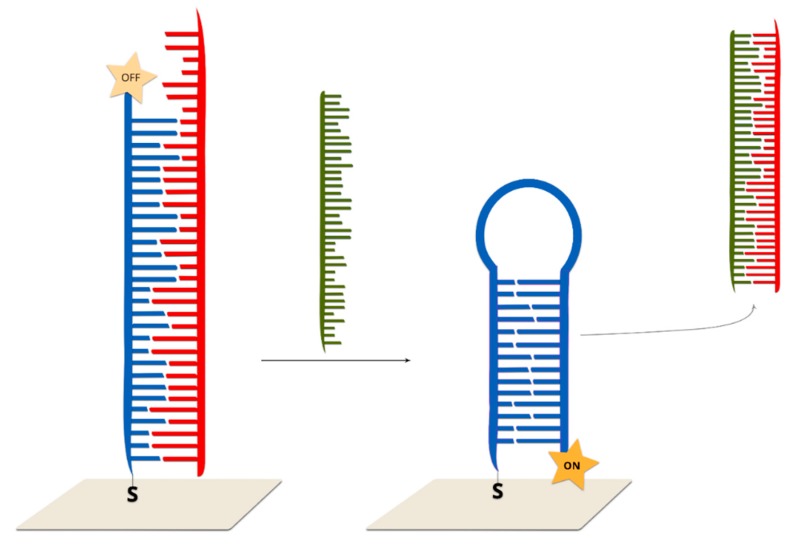
General scheme of hairpin off–on sensor: blue is capture DNA immobilized on metal surface labelled with RR, red is a “placeholder” DNA, and green is the target DNA (for example, mutated DNA from a clinical sample).

In such sensors, double-stranded DNA (the second strand prevents the formation of a hairpin configuration) is immobilized on the surface of a plasmonic structure. The addition of the target DNA which hybridizes with one of the strands (the one not connected with surface via a covalent bond) causes the formation of a stem–loop configuration of the DNA strand immobilized on the surface. As a consequence, the Raman reporter is moved to within close proximity of the plasmonic surface and a strong SERS signal is generated [8] (Figure 13).

The first example of a hairpin off-on system was reported by Wang and co-workers [8]. As the SERS active substrate, Au@Ag nanostars were applied due to the high enhancement of the electromagnetic field generated by such systems. In the first step, DNA probe strands with Raman labels are immobilized on the metallic surfaces via thiol bonds. A single-strand DNA placeholder was added, which led to partial hybridization. As a result, the Raman reporter molecule was separated from the metallic surfaces. Upon the addition of the target complementary sequences, the placeholder strand left the metallic surface. The target DNA was bonded with the toehold region and then began displacing the DNA probe from the placeholder via branch migration. As a result, the placeholder was released from the nanoparticle surfaces. After hybridization, a stem–loop structure was formed and, as a result, the Raman dye became located in close proximity to the metallic surface, and, hence, a strong SERS signal was observed. The SERS intensity of the bands of the Raman reporter decreased with increasing numbers of mismatched bases. For example, in the case of a one-base mismatch, the SERS intensity was reduced by 30% in comparison to the perfectly matched sequence. The limit of detection for the described method is extremely low and is estimated as 0.1 nM.

**Figure 14:**
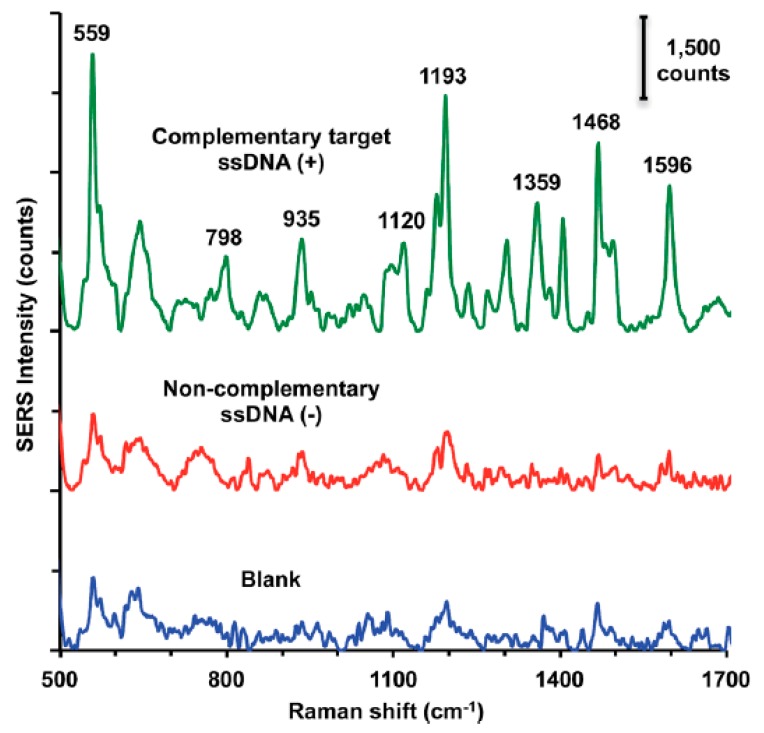
Recorded SERS spectra of: blank samples (blue line), non-complementary ssDNA (red line) and complementary target ssDNA samples (green line). Reprinted from reference [116] with permission from Royal Society of Chemistry. Copyright 2014 RSC.

In a similar manner, it is possible to detect dengue fever DNA [116]. In the absence of complementary target DNA, the Raman reporter molecule and placeholder maintain partial duplex structures, and, hence, the dye was located away from the metallic surface. Upon the addition of complementary target DNA, hybridization with the placeholder occurred and a stem–loop structure was created. As a result, the Raman dyes were brought into close proximity to the metallic nanostructures, inducing a strong SERS signal. For the blank samples and non-complementary DNA samples, SERS intensities were low, and the same as before the addition of the samples. This method is extremely sensitive and requires very small aliquots of samples. The concentrations of targeted DNA were 0.1, 0.4 and 1.0 µM. The sample volume was maintained at 30 µL, and so the absolute amounts of targeted DNA in the samples were 3, 12 and 30 picomoles. The limit of detection was estimated as 6 attomoles. Figure 14 showed SERS spectra in the case of the presence and absence of complimentary target DNA.

#### 2.2.5. Other Examples

There are also examples of the label-detection of DNA involving only two DNA strands. For example, peptide nucleic acids (PNAs) can be used as capture DNA, creating a negatively charged surface during hybridization with the target DNA, which can easily interact with the positively charged silver nanoparticles, later labelled with reporter molecules [117]. Interestingly, the nanoparticles and reporter molecules did not bond to the system containing non-complimentary DNA. The second advantage of the use of PNAs in a DNA sensor is the fact that, in contrast to DNA, PNAs do not show an affinity for an inorganic linker, Zr^4+^, because of the absence of phosphate groups in their structure [118]. Therefore, Fang et al. created a detection model consisting of PNAs immobilized on silicon nanostructures coated with gold–silver, target DNA and Zr^4+^, providing a limit of detection at the picomolar level.

A low-cost platform consisting of hierarchical Si nanowires and microscale pyramids was used to detect Epstein–Barr virus (EBV) DNA [119]. Probe DNA was immobilized on this surface while smaller AgNPs were modified with 4-MBA and EBV target DNA. After hybridization, SERS spectrum was collected. The presented platform is dual-function—it is also able to isolate carcinoma circulating tumor cells.

Graphene oxide–silver nanoparticles can also be used as an SERS platform [120] after the immobilization of thiolated DNA, forming a hybrid with the target DNA on their surface and, similarly to the previous example, being covered with silver NPs labelled with an RR. Moreover, this sensor has been applied for multiplexed DNA detection.

The described procedures look very uncomplicated compared to the majority of experimental systems, and might, therefore, be taken into consideration in studies on different DNA mutations.

## 3. Summary, Conclusions and Future Prospects

Much research effort is being put into the development of techniques enabling the detection of some DNA mutations, because the identification of such mutations is very useful in the early diagnosis of genetic disorders, and can influence clinical decisions. Nowadays, the identification of mutated DNA in clinical samples is mainly carried out using polymerase chain reactions and modifications thereof (for example, a quantitative polymerase chain reaction) as well next-generation sequencing. Because of the great practical importance of detecting certain kinds of mutated DNA, many research groups are trying to develop new methods for analyses that would be cheaper and/or would provide results in a significantly shorter period of time. One of these very promising techniques, which can be used to detect mutated DNA, is SERS. With the use of this technique, it is possible to detect even a single molecule of labelled DNA [121], outclassing practically all other analytical techniques employed in DNA detection.

Usually, the majority of SERS DNA sensors being developed nowadays utilize what are known as Raman reporters (chromophores having an exceptionally large cross section for Raman scattering). In such sensors, when a DNA fragment with a given sentence is present in the analyzed sample, it induces the movement of the Raman reporter into close proximity with the plasmonic nanostructure, or a movement which significantly increases the distance between the Raman reporter and the SERS substrate. This movement induces a very large change in the intensity of the measured SERS signal of the Raman reporter, and the detection of the DNA fragment with a sentence under investigation is based on a determination of the intensity of the SERS bands of the Raman reporter. In some other DNA SERS sensors, the detection of a specific DNA is based on a determination of the SERS intensity of bands, due to the vibrations of certain moieties of adsorbed DNA or the intensity of spectral features due to the vibrations of moieties present in the linkage layer through which DNA is attached to the plasmonic structure. In this review article, we presented only selected Raman approaches used to detect mutated DNA. In November 2019, in the database Scopus, the combination of two keywords “Raman” and “DNA” gives about 4000 hits. Moreover, one can easily propose many modifications of the techniques described above, for example, by utilizing nonlinear SERS, that is, surface-enhanced hyper Raman scattering (SEHRS) instead of classic SERS spectroscopy. The methods described above, therefore, should be treated as the methods which the authors of this contribution think are the most promising.

Like any analytical method, SERS spectroscopy has some disadvantages. Firstly, the cost of a Raman spectrometer is rather high and carrying out Raman measurements requires service from well-trained staff. One of major obstacle to overcome before a clinical implementation of SERS-based DNA sensors consists of the lack of reproducibility common in SERS signals. Due to the resonance condition, the intensity of the measured SERS signal is sensitive to the nanoparticles size and shape and, consequently, a little variation in the morphology of nanoparticles causes SERS signal changes. This effect greatly hampers the application of SERS spectroscopy for clinical samples analysis. Additionally, due to the high local electromagnetic field, enhanced organic molecules, like DNA, RNA or proteins, could be burned under laser beam impact. In such a case, the additional peak from amorphous carbon appears on the spectrum, which makes analysis difficult. Typically, plasmonic substrates are composed from plasmonic metals like gold, silver or copper. In some cases, a direct interaction between analyte and metallic surfaces could lead to a change in their structure. One solution to this problem was proposed by Li and co-workers in 2010, where metallic cores were coated by nanoscale layers of inert oxides like silica or alumina, which served to prevent nanoparticle agglomeration in addition to hindering direct contact between the analyte molecules and the metallic surfaces [122].

Unfortunately, as far as we know, no single SERS DNA sensors have been applied in clinical practice to date. This is mainly due to the still insufficient sensitivity of SERS DNA sensors. In real clinical samples used to detect mutated DNA (for example, samples of blood used to detect a circulating free tumor deoxyribonucleic acid), usually only a very small amount of the alleles present in the sample (about 0.1%–0.01%) are mutated. This often means that the difference between the Raman signal generated in experiments with samples containing such a small amount of mutated DNA and in samples without mutated DNA is very small. In order to incorporate SERS DNA sensors in actual clinical practice, the development of new, highly active, stable and very reproducible plasmonic substrates for SERS measurements is necessary. Therefore, in our opinion, in addition to the development of new strategies for the SERS detection of mutated DNA, a significant amount of basic studies on the development of new materials for SERS measurements must be conducted, especially because a combination of PCR and SERS provides very successful results for multiplexed DNA detection in real samples [123,124].

## Figures and Tables

**Table 1:** Comparison of currently used methods of molecular biology and surface-enhanced Ramen spectroscopy (SERS) for detecting mutations in DNA.

LP	Method	Principle of the Method	Sensitivity	Specificity *	Cost Per Reaction (USD)	Sample Preparation	Method Complexity	Main Application
1	allele-specific PCR	amplification refractory mutation system	1%–10%	+	1	hours	low	germline and somatic mutation genotyping
2	qPCR	fluorescently labeled probes	1%–5%	+++	2	hours	very low	germline and somatic mutation genotyping, gene expression, copy number
3	PCR–HRM	detecting small differences in PCR amplicon melting (dissociation) curves	1%–5%	++	2	hours	very low	germline and somatic mutation genotyping
4	Sanger Sequencing	fluorescently labeled nucleotides	10%–20%	++++	5	day	high	low frequency somatic mutation detection (liquid biopsy), very precise copy number detection
5	ddPCR	fluorescently labeled probes and emultion PCR	0.01%–1%	++++	3	hours	medium	low frequency somatic mutation detection (liquid biopsy), very precise copy number detection
6	NGS	massively paralelled sequencing	0.1%–5%	+++++	250	1–2 weeks	very high	low frequency somatic mutations detection (liquid biopsy), panel sequencing (10–400), whole genome sequencing
7	SERS	measurement of the Raman spectrum of Raman reporter	0.001%–1%	++++	n.a	hours	very low	germline and somatic mutation genotyping

+ Low specificity; +++ average specificity; +++++ highest specificity. * Methods' specificity is estimated on a 5-point scale where + is the lowest and +++++ is the highest specificity.
